# Examining the feasibility and characteristics of realistic weight management support for patients: Focus groups with rural, micropolitan, and metropolitan primary care providers

**DOI:** 10.1016/j.pmedr.2021.101390

**Published:** 2021-04-30

**Authors:** Gwenndolyn C. Porter, Robert Schwab, Jennie L. Hill, Todd Bartee, Kate A. Heelan, Tzeyu L. Michaud, Paul A. Estabrooks

**Affiliations:** a Deaprtment of Health Promotion, College of Public Health, University of Nebraska Medical Center, United States; b Department of Kinesiology and Sport Sciences, University of Nebraska at Kearney, United States; c Center for Reducing Health Disparities, College of Public Health, University of Nebraska Medical Center, United States

**Keywords:** PARIHS, Implementation, Primary care, Weight management

## Abstract

The purpose of this investigation was to understand perspectives of physicians, nurses, and staff regarding the feasibility of implementing an evidence-based weight management program to support primary care practice. An exploratory aim was to examine differences in responses based on the clinic location. Ten focus groups were conducted with primary care staff from rural, micropolitan, and metropolitan clinics. The Promoting Action on Research in Health Services (PARIHS) framework was used to inform the interview guide. Transcripts were reviewed to identify common themes among PARIHS constructs (evidence, context, and facilitation). Presence of comorbidities (e.g., diabetes, hypertension) were typical prompts for provider-led discussions about patient weight. Metropolitan clinics reported the availability of health coaching, diabetes education, or dietician consultation, but no clinic reported offering a comprehensive weight management program. Participants agreed it is possible to implement a weight management program through primary care, but cited potential facilitation challenges such as costs, clinic resources, and individual patient barriers. More enthusiasm arose for a referral program with patient tracking. Program characteristics such as proven efficacy, individual tailoring, program accessibility, and patient feedback to the providers were desired. Rural focus group participants reported unique barriers (lack of local resources) and facilitators (more flexibility in practice changes) to weight management when compared to metropolitan and micropolitan focus groups. Primary care staff are interested in weight management solutions for their patients and would prefer an evidence-based program to which they could refer patients, receive feedback on patient progress, and sustainably include as part of their regular services.

## 1 Introduction

Primary care practices have been identified as a potential location for intensive weight management interventions (WMI) to reduce obesity (Katzmarzyk et al., 2020, Wadden et al., 2020, Tronieri et al., 2019). This may be particularly attractive in rural areas where resources for promoting healthful eating and physical activity are scarce, obesity rates are high (Ogden et al., 2014, Perri et al., 1993), and primary care clinics are often the only available resources to support weight management (Phillips et al., 2014). Indeed, primary care may provide an opportunity to develop practical and sustainable systems-based approaches to address obesity (Glasgow et al., 2003, Glasgow et al., 2004) by 1) leveraging the patient-provider relationship to support program initiation and retention, 2) engaging organizational decision-makers, such as clinic medical directors and office administrators, to improve sustainability (Huang et al., 2011), and 3) using the human and technological resources of the primary care system to implement manageable change (Williams and Hummelbrunner, 2019). While there is growing evidence in the effectiveness of intensive WMI implemented through primary care, few have been systematically and consistently translated into typical clinical practice or community services (Estabrooks and Russell, 2006, Akers et al., 2010, Green et al., 2009).

The lack of translation of evidence-based WMI into primary care settings could be related to the lack of provider time to critically review the literature and identify an appropriate intervention, a mismatch between infrastructure and program intensity, and a lack of support in the initial adoption and implementation of a new intervention. Furthermore, issues related to the research to practice translation may also differ between rural and urban primary care settings where the availability of local resources differs significantly. The purpose of this research was to understand preliminary feasibility of implementing an evidence-based WMI through primary care, concentrating on factors related to the patient (e.g. patient identification and engagement, weight loss initiation and maintenance), clinical adoption (e.g. scalability, clinical uptake), and sustainability, all of which are key for the translation of evidence-based interventions into practice (Estabrooks and Russell, 2006, Leeman et al., 2017, Leeman et al., 2015, Chorpita et al., 2005). Secondary aims were to explore the successes and challenges of weight management programming that may already be present in clinics and to explore differences among clinics serving rural/micropolitan (cities with a population < 50,000) and metropolitan areas.

## 2 Methods

Ten focus groups were conducted with staff from primary care clinics of the Nebraska Practice-Based Research Network (PBRN) between November 2016 and October 2017. Clinic managers and/or head physicians of each clinic were contacted by the physician leader of the PBRN and asked if they and their staff would be willing to participate. Focus groups were grouped by clinic site to facilitate candid discussion among individuals who were familiar with one another, and to discern the context – within a given clinic – related to supporting patient weight management. We continued to recruit clinics until focus group responses reached saturation. All contacted clinics agreed to participate and reflected approximately two thirds of PBRN member clinics (i.e., 10 out of 15 clinics). Four clinics were located in rural areas (<10,000 residents), one clinic was located in a micropolitan area (urban core of at least 10,000, but less than 50,000 residents) (OMB, 2010) and the remaining five participating clinics were in a metropolitan area (468,262 population; U.S. Census Bureau). Of note, four of the five clinics located in a metropolitan area were members of the same health system. All other clinics came from different health systems.

### 2.1 Participants & conceptual framework

A total of 51 individuals participated in the 10 focus groups, with an average of five participants in each focus group (range: n = 2–12). Typically, one to two physicians and one to two nurses from each clinic participated, in addition to other personnel (Table 1). Table 2 provides an overview of the semi-structured interview guide and questions aligned with the Promoting Action on Research in Health Services (PARIHS) framework (Kitson et al., 1998, Stetler et al., 2011). This conceptual framework consists of three interacting elements – evidence, context, and facilitation – that influence the adoption and implementation of evidence-based practices (Rycroft-Malone, 2004). These elements and their sub-elements were used to create prompting questions regarding the feasibility of implementing a WMI through the clinic – including patient screening and referral, WMI delivery, clinical adoption, and sustainability. Questions related to current practices and structural characteristics were included to gain a better understanding of local practice information, context, and culture of weight management in Great Plains primary care. Focus groups concluded with a brief presentation of three specific evidence-based WMI (Appendix A) (Wyatt et al., 2008, Perri et al., 2008, Almeida et al., 2014). These WMIs were selected from a systematic review of rural WMI (Porter et al., 2019) for their demonstrated effectiveness, potential for delivery in a rural area, and for their variety of delivery methods, so as to gain an understanding of clinic staff’s perceptions of a wide variety of program characteristics. This study was approved by the Institutional Review Board at the University of Nebraska Medical Center and informed consent was obtained from all participants.

**Table 1:** Participant characteristics.

Titles	N	% Female	Mean (SD) years in clinical role
Physician	15	31%	12.0 (10.8)
Nurse (RN, LPN, Nurse manager)	11	100%	8.6 (7.5)
Clinic manager, Administrator	9	100%	11.6 (9.9)
Physician Assistant, Nurse Practitioner	7	100%	8.5 (6.0)
Other clinic staff	7	89%	7.9 (8.9)
Health coach, coordinator	2	100%	15 (11.0)
Total	51	76%	10.4 (9.5)

Note: Other clinic staff included medical assistants, a phlebotomist, a pharmacist, and behavioral health staff.

**Table 2:** PARIHS framework constructs and example interview guide items.

Evidence	Context	Facilitation
• *Research and published guidelines:* What evidence-base will you need to consider when using a weight loss program in an ongoing way at your facility? • *Clinical experience and perceptions:* Right now, in your clinic, what kind of activities or programs do you have in place that address excess body weight with your patients? • *Patient experiences, needs and preferences:* How important do you think it is to your patients to have weight loss programs available to them? • *Local practice information*: Have you come up with any solutions to help your patients address challenges to weight loss? • *Characteristics of the targeted EBP:* Besides scientific evidence, what other factors need to be considered when deciding to implement a weight loss program?	• *Leadership support:* How would the leaders in your organization prioritize a weight loss intervention for your patients? • *Culture:* How well would addressing obesity at your clinic fit with the other services you provide? • *Evaluation capabilities:* Can you tell me how your clinic currently tracks patient weight or BMI? • *Receptivity to the targeted innovation/change:* If your clinic decides to implement a more focused approach to addressing weight loss, what challenges do you see arising?	• *Role of facilitator*: If you decided to make the weight loss program a permanent fixture at your facility, who would be the person in your clinic to champion a program like this? • *Role skills and attributes*: What are some of the skills a facilitator would need to get a weight loss program up and running at your site? • *Other implementation interventions*: What kind of changes would your organization need to make to the roles for staff so that a weight loss program could fit with the other services you provide?

### 2.2 Data analysis

Focus groups were audio recorded and transcribed verbatim, separated into meaning units, and assigned a descriptive code by two to three independent coders (Elo and Kyngäs, 2008, Graneheim and Lundman, 2004). Once completed, each coding pair/trio met to reconcile any coding differences by consensus. Additional coders were consulted if a consensus could not be reached. Common meaning units across questions were deductively grouped together by codes and a nested hierarchy of meaning units were grouped into themes relevant to the PARIHS framework constructs (i.e., context, facilitation, and evidence). Table 3 provides a sampling of quotes and key takeaways related to these constructs.

**Table 3:** Example quotes and key takeaways for each PARIHS framework construct.

Theme	*Example Quotes*	Key takeaways
**Context**		
Weight measurement & evaluation	*“The EMR has several different ways to track [weight] all the time.” (Physician, metropolitan clinic).* *And for those that have high BMIs, when you click on the quality measures, it says, ‘elevated BMI’ and you have to document - did you have a discussion with the patient about weight, and that sort of thing. So, you have to do that, I think, once a year. It’s new so we’re not sure. I just know if I hit that button and it says, ‘has it been addressed?’ then I have to address it.” (Physician, metropolitan clinic).* *“The problem with the goal-based system is that it has to be addressed theoretically by the provider at the visit. So, all we are doing is adding more things for me to do during the visit. I’m going to do it the same way that I’ve always done it. I don’t have more time in my day, and you just gave me more to do.” (Physician, metropolitan clinic).*	All clinics track weight and BMI in some capacity EMR capacity to nudge providers to action due to elevated BMI varies greatly EMR alerts due to elevated BMI may lead to “click fatigue” and lack of action Provider time to address elevated BMI is limited
Culture – Weight management for patients	*“[I will discuss weight] with someone who has comorbidities and obviously, you have diabetes, hypertension, you have a heart problem, something you can link [to weight].” (Physician, rural clinic)* *So, I'll use my diabetic population right off the bat. So somebody who is diabetic, hypertensive, and especially the one thing I always look for - that have osteoarthritis symptoms…so when you see a few things going together, and it's one of those things like hey, okay, you just complained to me about your chronic knee pain, you're a hypertensive diabetic and your BMI is 32. Well I start building that whole picture…and try to point out to the patient, your weight could have a lot to do with your knee, I guarantee it has a lot to do with you insulin resistance, it has a ton to do with your hypertension… [We talk] about what their goals should be for aerobic exercise … I try to always mention strategies, whether it be portion control or for some people talking with them about their vices a little bit, and to me it's always about trying to figure out … what's the driver behind their weight problems. Is it inactivity? Is it caloric intake? Is it a combination of both? Is it other chronic medical conditions? (Physician, metropolitan clinic)* *“I’ll go in and start to discuss with them what they’d like to change, what their goals would be. I make my note. Then she [health coach] follows-up with that. She has time to give them a call, have them come back in, do another weight, check on their [goals], basically lets them know that we’re still here for them and that they can follow-up with us. That does help.” (Physician, rural clinic)* *“I always ask about [patient’s] goals. Ask their motivation…if they have any intention of doing anything to lose weight” (Nurse, micropolitan clinic)* *“The providers will be doing the majority of putting those goals in [the EMR], but (the goals) have to be also set by the patient. That’s where the care coordination will be important in identifying the action that they can take based on the goals that the patient sets.” (Health coordinator, metropolitan clinic)* *[Weight loss medication] an adjunct. My rule is, other than the blood pressure and heart rate have to be fine, and if they want it they have to be exercising and eating healthy. It's not a substitute for doing the things that we ask them to do, but it's an adjunct. (Physician, rural clinic)* *Maybe 50% of [patients] stick with it (weight loss mediation) long-term. (Health coordinator, metropolitan clinic) When they realize that the pill isn’t going to be magic, they didn’t lose their 20 lb the first month, [they stop taking the medication]. (Health coach, metropolitan clinic) When they go off of it and they haven’t made the lifestyle changes, they gain it all back. (Health coordinator, metropolitan clinic)*	Visit type and existing comorbidities were common triggers used by provider to discuss weight Providers have strong attitudes related to the need to address obesity and its underlying causes Providers perceive that a balance is needed, and patients need to identify goals and demonstrate some motivation for change Provider use of goal-setting varies across clinics; patient access to goals varies Providers have little enthusiasm for prescribing weight loss medication – used as an adjunct to lifestyle recommendations Primary concerns related to medications is that the weight loss is often well below patient expectations and, as a result, they discontinue use. This issue was not considered as significant for weight loss through behavioral interventions—though providers did express that weight loss maintenance is a challenge
Leaderships support for patient weight management	*“In terms of tracking (weight), there is no other reports run on the routine basis. Providers are just encouraged to address it at every visit…but there is no formal way.” (Physician, metropolitan clinic)* *“It’s not required (that we have patient weight discussions), but we’re highly encouraged to do it.” (Physician, metropolitan clinic)* *“I think there would be pretty minimal issues…If there was a good, well-thought-out plan as to how it (weight loss program) would be implemented into the EHR system and the workflow.” (Physician, metropolitan clinic)*	Providers encouraged to discuss weight with patients, though there is no formal structure for such discussions
**Facilitation**		
Feasibility of implementing a weight management program through primary care	*“The big factor going forward is going to be how people get reimbursed because obviously [clinic staff] can’t be taking out large chunks of their day and not get paid for it.” (Nurse, micropolitan clinic)* *“We don’t have the extra manpower sitting around to do it,” (Nurse, metropolitan clinic)* *“[Prioritization of a new weight management program] would fall to the very bottom. We’re just trying to keep up with what’s required on a daily basis. So, to add another program would be a stress.” (Staffer, metropolitan clinic)* *“I think we have to look at things that have value and things that we can do. We’re doing a lot of things. So, that's why there's some hesitation here. Do we have the capacity to do that?” (Physician, rural clinic)* *“Maybe if they attempted previously (to lose weight), use Weight Watchers, some sort of exercise, if they have attempted before.” (Physician, micropolitan clinic)* *“[Insurance coverage] is my only fear of a separate entity program,” (Physician, rural clinic)*	Clinics would need to weigh the value of a new program and clinical capacity to implement it Clinics have significant hesitations about their capacity to implement a program in-clinic External programs were favored, hover concerns of reimbursement and insurance coverage were raised
**Evidence**		
Clinical experience and perceptions	*“If we had the support to do it. I think all of us would like to offer additional support for weight loss to all our patients. It’s just finding the personnel or the time to do that. Or the right program.” (Staffer, metropolitan clinic)* *“It’s the accountability. If you have somebody to keep you accountable then you’ll be successful.” (Physician, metropolitan clinic)*	Primary care staff agree patients would appreciate having a weight loss program available Primary care staff believe patient accountability is necessary
Local practice information	*“*…*definitely they (patients) would appreciate having something, a program in place that they could get some education, and some exercise. And more consistency across the board because everybody (providers) does things differently. Everybody has different tips you know.” (Physician, rural clinic)*	Consistency with a referral program would be appreciated by providers
Patient experience, needs, preferences	*“…(patients) don’t want to acknowledge that they are overweight but as soon as you mention obesity and weight loss it’s like that wall shoots up and they just get defensive, like ‘no, no I’m not obese, I don’t know why you’re saying that,’” (Nurse, rural clinic)* *“There are also a lot of people that don’t see their weight as a problem,” (Staffer, metropolitan clinic)* *“It’s very typical in the community to work ten-, twelve-hour shifts, like I said, five, six, seven days a week.” (Physician, rural clinic) “Sixty hours per week is pretty normal in this town.” (Physician, rural clinic) “So by the time you get home, and you want time for your family, and you want time to rest, there’s really not a lot of other time that you really want to spend doing this type of activity.” (Physician, rural clinic)*	Individual patient barriers including perception of weight, the food environment, transportation, occupation, and telephone/internet access may hinder patient participation in a weight management program
Characteristics of the targeted evidence-based practice	*“… if we would refer, the communication back how the patient is doing, that would be huge. That would be huge to make it successful. At least in the 20-minute slots we could say ‘Hey, we heard you were doing great. Keep up the great work. We’ll keep following along.’ I think that could help it be really successful.” (Nurse, metropolitan clinic)* *“I think so much of it is patient dependent. I think the home program would be good for someone who doesn’t have transportation. But I also think there’s a lot gained from a group program.” (Staffer, metropolitan clinic)* *“[the program] would have to be individual-based.” (Business manager, rural clinic)*	Preferred characteristics of a weight management program included patient and provider feedback on progress, maintaining patient accountability Program characteristics that would influence a decision to adopt a weight management program included cost, program efficacy, program objectives, frequency of program interactions, availability of program offerings, and delivery method.

## 3 Results

### 3.1 Context—Current practice in primary care

#### 3.1.1 Weight measurement & evaluation

All clinics measured and tracked weight at every patient visit using an electronic medical record (EMR) system, which automatically calculated patient BMI. Most EMR systems alerted clinic staff if BMI was in the obese range or if there had been a significant change since the patient’s last visit (e.g. change in font color, a pop-up window, an automatically generated email to the provider). Some systems had a BMI quality measure, which included forced entry fields for the provider to document if a high BMI (>29.9 kg/m^2^) was addressed during that patient visit. Participants acknowledged the capabilities of their EMR systems, but responses were inconsistent across clinics regarding if and how EMR alerts or quality measures prompted action during a patient encounter. However, there was consistency on the issue of “click fatigue,” (Collier, 2018) particularly related to forced pop-ups and check boxes related to weight, BMI, and many other clinical notifications. Participants stated that although well intended, the weight/BMI quality measure did not always have the intended effect in practice. As one physician noted, “I don’t have more time in my day, and you just gave me more to do.” No differences were observed by clinic locations regarding weight and BMI tracking, EMR alerts, or “click fatigue”.

#### 3.1.2 Culture of weight management support for patients

Three major themes emerged regarding current clinical culture surrounding weight management – providers delivered health education, medication, and referral to an outside program. No participants reported having a formal WMI available at their clinic, regardless of location.

Provider health education included informal discussions led by the provider during a patient visit. Two triggers were identified for these conversations: visit type and patient comorbidities. Patient weight was generally addressed only at health maintenance visits or when weight was pertinent to the reason for the visit, regardless if an EMR prompt was given. For example, one physician described how they initiate a weight discussion at a health maintenance visit in this way: “So somebody who is diabetic, hypertensive, and especially the one thing I always look for - that have osteoarthritis symptoms…so when you see a few things going together, and it's one of those things like hey, okay, you just complained to me about your chronic knee pain, you're a hypertensive diabetic and your BMI is 32. Well I start building that whole picture.” Similarly, most physicians reported using weight-related comorbidities as cues to introduce a discussion about patient weight and to illustrate to patients how weight impacts those comorbidities. Physicians reported limited use of BMI during patient discussions – typically only when the patient understood what BMI represented or when it could be used as a “road map” to draw a patient’s attention to their weight as a concern.

The focus of these discussions generally centered on patient eating and physical activity behaviors and goal setting. Some providers used an informal, conversational approach to patient goal setting and overcoming barriers, while others used a formal goal setting tool that is built into the EMR system, which, in some cases, could be viewable and editable by the patient. In rare cases, physician-initiated goal setting prompted further action by clinical staff such as a health coach or care coordinator. Other clinic staff also described how they talked with patients about their health behaviors and how those behaviors affect weight. These interactions happened during the patient visit or were scheduled as a follow-up appointment with the specific staff member (e.g. health coach, coordinator). Most participants also reported they had referred their patients to a dietician. In rural/micropolitan clinics, these dieticians were employed by local grocery stores, while metropolitan clinics mentioned nutritionists or dieticians employed by the clinic or their parent healthcare organization. Metropolitan clinic participants mentioned that their nutritionists/dieticians were often covering multiple clinics at a time, which made it difficult to schedule patients and nearly impossible to monitor patient progress after the referral was made. One metropolitan clinic staff participant said that their nutritionist was the “highest to have a no-show” out of all the providers at that clinic, “probably because she’s (nutritionist) forced to schedule [appointments] so far out.”

Prescription weight loss medication and referral to bariatric surgery were reported as additional methods to treat overweight/obesity among patients. Providers rarely used medication as monotherapy, but all reported prescribing weight loss medication to some degree, primarily after a patient requested it. All participants reported short-term success of weight loss medication among highly motivated patients, but reported scarce long-term success and showed little enthusiasm for weight loss medication, presumably due to unrealistic patient expectations. As one health coordinator said “When they realize that the pill isn’t going to be magic, they didn’t lose their 20 lb the first month, they go off of it and they haven’t made the lifestyle changes, they gain it all back.” Participants reported long-term success among patients that were referred for bariatric surgery but noted this was only a small proportion of their patients. No differences were observed in responses from participants by clinic locations regarding provider health education prompts and content, care coordinator/health coach presence, or for weight loss medication use or surgery referral.

Finally, most participants utilized external weight loss resources when available, such as referring patients to local dieticians, healthy living or nutrition programs delivered through local YMCAs or medical centers, commercial programs such as Weight Watchers, and suggesting local physical activity resources. The availability and number of patient referrals to external resources was dependent upon clinic location – metropolitan and micropolitan clinic participants consistently cited more community resources than participants at rural clinics, who cited distance and transportation as major barriers to connect patients with weight loss resources. All focus group participants reported a lack of follow-up or monitoring of patient progress after they are referred to an external weight loss resource.

#### 3.1.3 Leadership support for patient weight management

The majority of participants reported being encouraged by clinical leadership to talk to patients about weight, but with no specific directives given. In terms of adopting a new process or program, some raised the concern that while clinic leadership may vocalize support for patient weight management efforts, funding such efforts may be a challenge. When asked if clinic leadership would support physicians referring patients to a WMI, one staff participant said, “I’m sure the answer would be yes to that, but whether or not the funding would follow that, that’s the question.” Differences emerged by clinic locations regarding program adoption. The rural clinics – many of which were single-standing clinics – reported having more freedom to adopt new programs and practices compared to metropolitan clinics – all of which were governed by large healthcare organizations.

### 3.2 Facilitation—feasibility of implementing a weight management program through primary care

While all participants agreed a WMI connected to primary care would be beneficial, significant concerns were raised regarding program implementation such as facilities, staff availability, and costs. Clinics from all areas expressed the need to weigh clinical capacity for implementation with the potential patient and clinical benefits before deciding to adopt a WMI.

All participants were more enthusiastic when asked to consider the feasibility of an external WMI to which clinics could refer patients and follow-up on progress. Patient identification and referral to an external WMI would likely occur through an EMR query of high weight or BMI, most participants noted. Discussions naturally moved from the ease of an EMR query to the challenge of gauging a patient’s motivation to participate. Potential challenges raised for external programs included program cost – particularly insurance coverage, staff time for patient identification, referral, and follow-up. Referral to external programs was generally recognized as a more feasible option than adopting and implementing a program in clinics, though as one physician noted insurance coverage “is my only fear of a separate entity program.”

When asked who would be responsible for championing a program should their clinic decide to adopt a WMI there was no consensus among clinics. Some participants suggested the idea of co-champions, and two participants from different clinics suggested sharing the responsibilities of program facilitation among all clinical staff. Most participants mentioned the importance of having buy-in from all clinical staff for program implementation and sustainability to be successful.

### 3.3 Evidence – experience, needs, and preferences for weight management options in primary care

#### 3.3.1 Clinical experience and perceptions

Very few participants reported instances of patients asking about lifestyle WMIs. However, there was consensus that both clinical staff and patients would appreciate if a WMI was available. One staff participant noted “If we had the support to do it. I think all of us would like to offer additional support for weight loss to all our patients. It’s just finding the personnel or the time to do that. Or the right program. “A theme of patient accountability emerged – the majority of participants mentioned the need for a program that will keep patients accountable, such as in-person, telephone, or digital counseling support.

#### 3.3.2 Local practice information & patient experience, needs, and preferences

Most participants believed their patients would have an interest in a lifestyle WMI, if it were available, but felt some patients’ expectations regarding weight loss and perception of obesity was misguided and may be a barrier to their participation in a WMI. Additional barriers such as the food environment, transportation, and telephone/internet access were cited by participants across clinic locations. Notably, the issue of the food environment was rooted in the comparatively higher costs of healthy foods when discussed at the metropolitan clinics [“You walk down the (grocery store) aisle and there’s a big thing of cheese balls…And it’s 5 bucks! Whereas one red pepper is 2 dollars.”] yet rooted in a lack of access to healthy meal options for the rural clinics [“It’s just like, what can you get at McDonalds? What can you get at Taco Bell? What can you get when you go to a ball game? Or what can you pack ahead of time?”] Another potential barrier unique to rural focus group participants was patients’ work schedule due to the high proportion of shift workers in rural areas. As one physician noted, “It’s very typical in the community to work ten-, twelve-hour shifts, like I said, five, six, seven days a week.”

#### 3.3.3 Characteristics of the targeted evidence-based program

##### 3.3.3.1 Comparison of three evidence-based interventions

Participants reviewed three evidence-based programs (see Appendix A) and were asked to consider which program might fit best with their clinics. The first program, Calcium Weighs-In, (Wyatt et al., 2008) was well liked for its simplicity, perceived ease of implementation, and short duration, which participants initially felt would contribute to greater patient interest. However, the focus on diet and provision of daily meals caused concerns about program trialability, potential effectiveness among their patients, and associated costs. The second program, TOURS, (Perri et al., 2008) was favored for its evidence of weight loss but was thought to be the least feasible to implement through primary care and the least likely to be successful due to the high number of in-person sessions. “I just don't see the longer programs and the more expensive programs working at all” (Staff, rural clinic). Finally, while participants had both positive and negative comments about each program, they responded most favorably to the third program – diaBEAT-it! (Almeida et al., 2014) – “I think the diaBEAT-it! one would be much more applicable and feasible from a population standpoint.” (Physician, metropolitan clinic) DiaBEAT-it! was favored for its potential for high reach (delivered via DVD and automated telephone calls) though some participants expressed concerns that the advantages of a digitally delivered program might come at the price of participant engagement. Statements such as “It’s too easy to ignore” (Physician, rural clinic) and, “I don’t know that people would answer the phone calls” (Nurse, rural clinic) demonstrated these concerns.

## 4 Discussion

Within a systems-based approach to implementation, primary care practices have the structure to provide practical and sustainable methods to address weight management (Glasgow et al., 2003, Glasgow et al., 2004) through clearly defined clinical roles and intended audience. Our findings suggest a variety of individuals, or multiple individuals, within primary care clinics in the Great Plains could serve as program champions, acknowledging the need for all staff to support the program to achieve success.

Facilitation for a referral strategy appeared to receive more endorsement from the clinical staff due to the ease of implementation and evaluation of effectiveness based on number of patients that are referred. Regardless of the facilitation strategy and personnel, consistent with other literature, it is unlikely that either the implementation of an evidence-based WMI or referral strategy would be adopted without some integration of required tasks with personnel job responsibilities and duties (Hunter et al., 2020). A referral program was perceived to have a relative advantage over an in-house WMI for reasons of lack of time during patient visits and competing clinical demands, consistent with the literature (McKenna et al., 2004). The underlying reason for this was because a referral process could be completed quickly during patient visits and would not provide much disruption to competing clinical demands (higher compatibility, lower complexity). However, a discordance between this infrastructure and clinical culture to address obesity existed in that regular prompts were seen as white noise rather than a cue to action (Ew and Grove, 2016).

Differences were observed among responses from participants by clinic locations. Consistent with the literature (Porter et al., 2019, Perri et al., 1984), clinical staff from metropolitan clinics reported more community resources available for weight management compared to micropolitan or rural area clinics. Participants’ perceptions of the root of individual patient barriers appeared to differ among rural and micropolitan (access, work schedule), and metropolitan patients (cost). Primary care staff reported few patients proactively asked about a WMI and made statements suggesting their overall patient panel was unmotivated to seek out a WMI through primary care. However, these perceptions of patient motivation may be underestimated, as previously documented (Befort et al., 2006).

These findings have significant implications for the potential implementation of WMI through primary care. Cost was consistently cited as a barrier to implementing interventions in clinical or community settings and was reinforced by our findings. Program costs, reimbursement concerns, and staffing costs related to time spent identifying and recruiting patients and implementing program activities are proximal barriers to adoption and implementation decisions. Low costs, technology-supported interventions provide a simple referral pathway for primary care practitioners and could help resolve these issues; specifically, it would alleviate the cost burden for clinics and participants. Contextual factors related to addressing obesity in primary care clearly include external barriers surrounding providers’ ability to provide the needed resources with limited opportunities for reimbursement of services. This underscores the need for policy change related to state and insurance coverage opportunities that can vary broadly from region to region. Indeed, even for scalable and technology-supported weight loss programs that primary care providers perceive to be a good fit for their settings are not currently eligible for reimbursement through Medicare or other insurance providers. Addressing these external factors from a public health perspective could greatly increase the potential uptake of such programs in primary care.

Additionally, establishing partnerships between primary care clinics and local community organizations has great potential to improve program reach, implementation, and sustainability, and may also help distribute the cost of implementing an evidence-based WMI in a successful and sustainable way (Ackermann and Marrero, 2007, Ackermann et al., 2008). Examples of facilitated community-primary care linkages for weight management exist and have been successful not only in patient weight loss but also in maintaining the clinical-community partnership following the research study (Jebb et al., 2011, Lavin et al., 2006). Additionally, primary care referral to a commercial program may provide a long-term cost-effective solution for patients at risk of weight-related comorbidities (Fuller et al., 2014). Undoubtably, challenges will arise at onset. However, our findings provide support that community programs are favorable options for primary care providers wishing to improve patient weight management and have the potential to be successful when strong cross-organizational partnerships and champions are involved (Estabrooks et al., 2019).

There are limitations to this study that warrant consideration. While we found saturation in responses, the degree to which these findings can generalize to primary care settings outside of our sample, especially outside the Great Plains, is unclear. Due to the great variability among some clinical characteristics, such as EMR systems and functionality, we caution extrapolating findings regarding structural characteristics of the clinics. We did not collect information on clinical characteristics, which may limit the robustness of our analysis. We did, however, compare responses from clinical staff of varying geographic areas. Our findings, in concert with the literature, have practical implications for clinical practice and research, particularly regarding sustainability of interventions delivered through primary care. Primary care clinics are interested in weight management solutions for their patients and would prefer an evidence-based program to which they could refer patients, receive feedback on patient progress, and continually offer as a sustainable part of their regular services. Facilitation of WMI delivered through primary care appears to rely heavily on buy-in from clinical staff and may be improved by instituting co-champions to facilitate implementation strategies. Establishing co-champions may also address the expressed desire for patient feedback with an integrated research-practice partnership model (Estabrooks et al., 2019), and address sustainability considerations.

## CRediT authorship contribution statement

**Gwenndolyn C. Porter:** Conceptualization, Methodology, Investigation, Writing - original draft, Writing - review & editing. **Robert Schwab:** Supervision, Investigation. **Jennie L. Hill:** Conceptualization, Methodology, Writing - review & editing. **Todd Bartee:** Supervision, Writing - review & editing. **Kate A. Heelan:** Supervision, Writing - review & editing. **Tzeyu L. Michaud:** Writing - review & editing. **Paul A. Estabrooks:** Conceptualization, Methodology, Investigation.

## Declaration of Competing Interest

The authors declare that they have no known competing financial interests or personal relationships that could have appeared to influence the work reported in this paper.
