# Supplementary material for: Examining the feasibility and characteristics of realistic weight management support for patients: Focus groups with rural, micropolitan, and metropolitan primary care providers
Source: Prev Med Rep. 2021 Apr 30;23:101390. doi: 10.1016/j.pmedr.2021.101390 (PMC8134728; doi:10.1016/j.pmedr.2021.101390)

Appendix A: Overview of three evidence-based programs reviewed and discussed by all focus group participants.


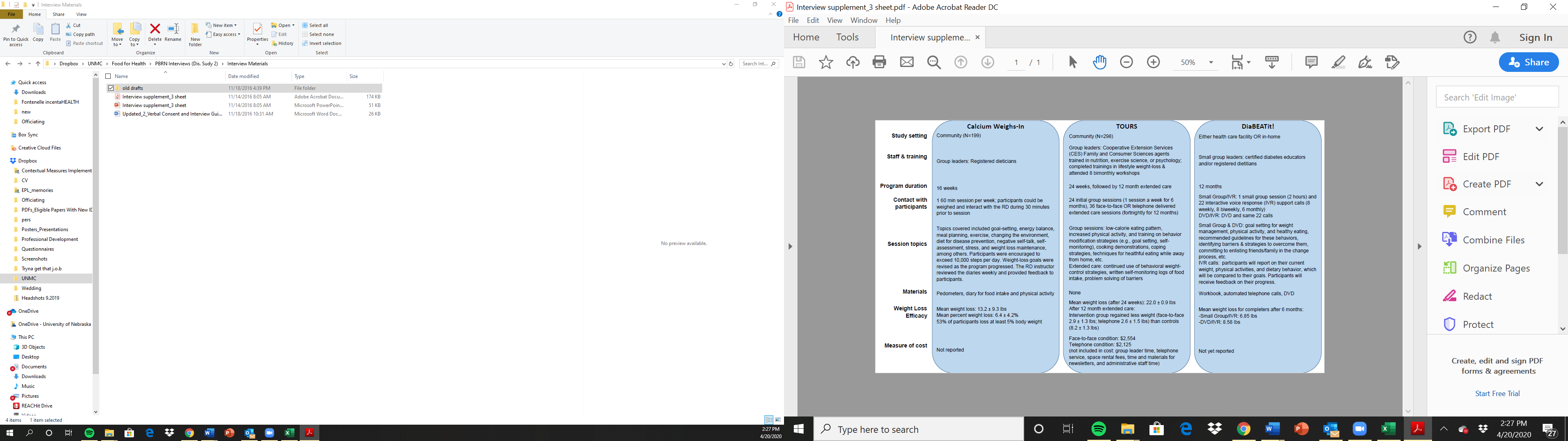

Supplement: Supplementary data 1 [file mmc1.docx]
